# The Oldest Evolutionary Lineage of *Trichoneura* Loew, 1850 (Diptera, Limoniidae) and the First Evidence of This Genus in Cretaceous Spanish Amber

**DOI:** 10.3390/insects12050411

**Published:** 2021-05-03

**Authors:** Iwona Kania-Kłosok, Wiesław Krzemiński, Katarzyna Kopeć, Antonio Arillo

**Affiliations:** 1 Department of Biotechnology, Institute of Biology and Biotechnology, University of Rzeszów, Zelwerowicza 4, 35-601 Rzeszów, Poland; 2 Institute of Systematics and Evolution of Animals, Polish Academy of Sciences, Sławkowska 17, 31-016 Kraków, Poland; wieslawk4@gmail.com (W.K.); k_slazyk@poczta.onet.pl (K.K.); 3 Departamento de Biodiversidad, Ecología y Evolución, Facultad de Biología, Universidad Complutense, 28040 Madrid, Spain; antonioarillo@gmail.com

**Keywords:** fossil insects, Cretaceous, Spanish amber, new species, new subgenus

## Abstract

**Simple Summary:**

A new established subgenus (genus *Trichoneura*, family Limoniidae) from Lower Cretaceous Spanish amber represents the oldest lineage of the genus and exhibits a unique morphology of hypopygium characterized by a huge lobe on the gonocoxite. The discovery of this new subgenus sheds new light on the chronostratigraphic distribution and diversity of the genus *Trichoneura* and the evolution of the Limoniidae. The is oldest known species of *Trichoneura* and is important for understanding the evolution of this group of insects.

**Abstract:**

A new subgenus *Cretalinea* subgen. nov. of *Trichoneura* (Diptera, Limoniidae) is established with one new species: *Trichoneura* (*Cretalinea*) *xavieri* subgen. et sp. nov. This is the first report of the genus *Trichoneura* in Spanish amber and the first record of the genus from the Lower Cretaceous period. The oldest described species of *Trichoneura* is compared with other species of the genus with particular reference to those known species from the Upper Cretaceous. A list and key of fossil species of *Trichoneura* are given.

## 1. Introduction

With regard to extant fauna, the genus *Trichoneura* Loew, 1850 [1] is sparse in its number of species and is found in three zoogeographical regions, primarily in the southern hemisphere. The genus *Trichoneura* is most rich in species in the Oriental region, where eight species of the genus have been recorded. Less abundantly they occur in the Afrotropical region (four species), and rarely in Australia/Oceania where only one species has been reported [2,3].

This genus is represented in recent fauna by three subgenera: *Ceratolimnobia* Alexander, 1920 [4] (two species), *Trichoneura* Loew, 1850 [1] (one species) and *Xipholimnobia* Alexander, 1921 [5] (ten species). In the fossil record only one subgenus, *Trichoneura* occurs (Table 1).

We only know five species from the fossil record, four from Eocene Baltic amber. Only one species was previously known from the Cretaceous period, from Upper Cretaceous Canadian amber [6] (Table 1). A representative of the genus *Trichoneura* has been found recently for the first time in Spanish Cretaceous amber and is described herein. It is also the oldest representative of the genus *Trichoneura*. Thanks to this new discovery, it was possible to shift the stratigraphic range of the genus *Trichoneura* from the late Cretaceous to the early Cretaceous. This new, peculiar species from amber of Spain described herein, provides evidence of the existence of *Trichoneura* flies in the early Cretaceous.

## 2. Material and Methods

**Figure 1:**
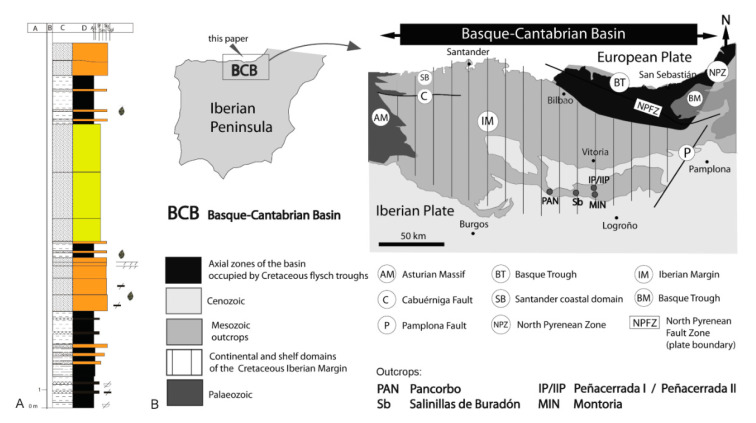
(**A**) Stratigraphic profile of the Peñacerrada I outcrop after [13], modified. (**B**) Geographical and geological setting with location of the studied section after [15] modified.

The study was based on material from Cretaceous Spanish amber. The specimen comes from the upper Albian amber-bearing deposit of Peñacerrada I (Basque—Cantabrian Basin, near the village of Moraza, Province of Burgos) (Figure 1). The specimen is deposited at the *Museo de Ciencias Naturales de Álava, (Vitoria, Spain)*.

The specimen was embedded in epoxy resin (EPO-TEK 301) [10,11] which allowed physical protection and optimal study in ventral, lateral and dorsal views.

The biological inclusion was examined with a Nikon (SMZ25) stereomicroscope, Nikon SMZ 1500 equipped with a Nikon DS–Fi1 camera. The measurements were taken with NIS–Elements D 3.0 software. The length of the discal cell was given from its posterior edge to the point of connection of vein m-m with vein M_3_. The length of hypopygium was measured from the posterior margin of tergite IX to the tip of the gonocoxite. The measurements were given only for undamaged structures, in millimeters (mm), and the length of scape, pedicel, flagellomeres and particular segments of palpus were given according to the pattern: antenna or palpus section number/length of this section, in millimeters. Drawings were made by tracing the photographs. The terminology, wing venation and male genitalia nomenclature followed that of [12,13,14].

## 3. Results

### 3.1. Systematic Paleontology

Order Diptera Linnaeus, 1758 [16]

Infraorder Tipulomorpha Rohdendorf, 1961 [17]

Family Limoniidae Speiser, 1909 [18]

Subfamily Limoniinae Speiser, 1909 [18]

Tribe Lechriini Alexander, 1927 [19]

Genus *Trichoneura* Loew, 1850 [1]

### 3.2. Cretalinea *subgen. nov.*

**Figure 2:**
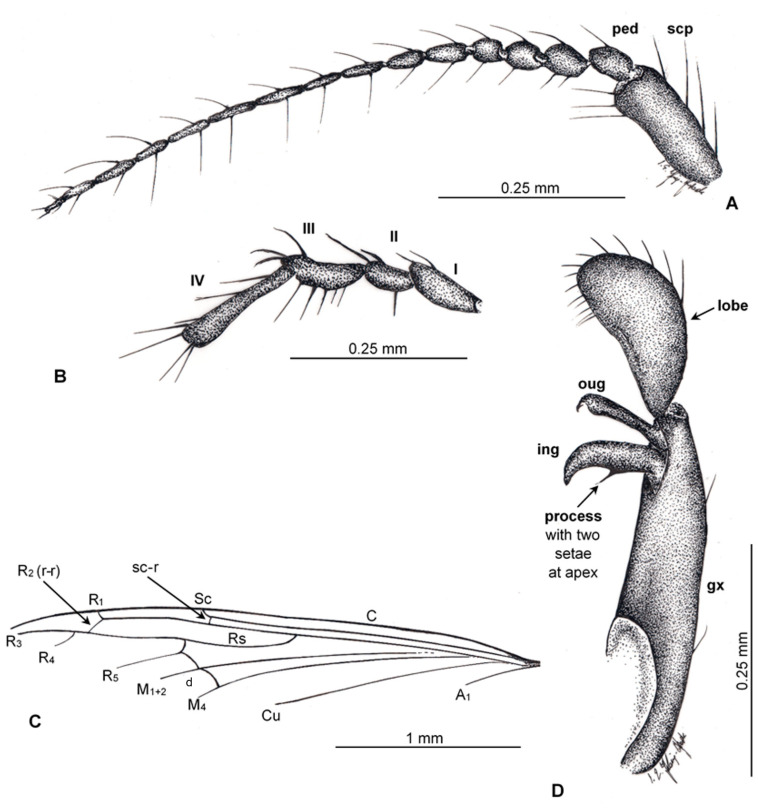
*Trichoneura* (*Cretalinea*) *xavieri* subgen. et sp. nov., holotype No. MCNA 9735 (male). (**A**) antenna; (**B**) palpus; (**C**) preserved of part of wing; (**D**) gonostyli and gonocoxites. Abbreviations: scp—scape; ped—pedicel; I–IV—palpomeres 1–4; ing—inner gonostylus; oug—outer gonostylus; gx—gonocoxite.

**Figure 3:**
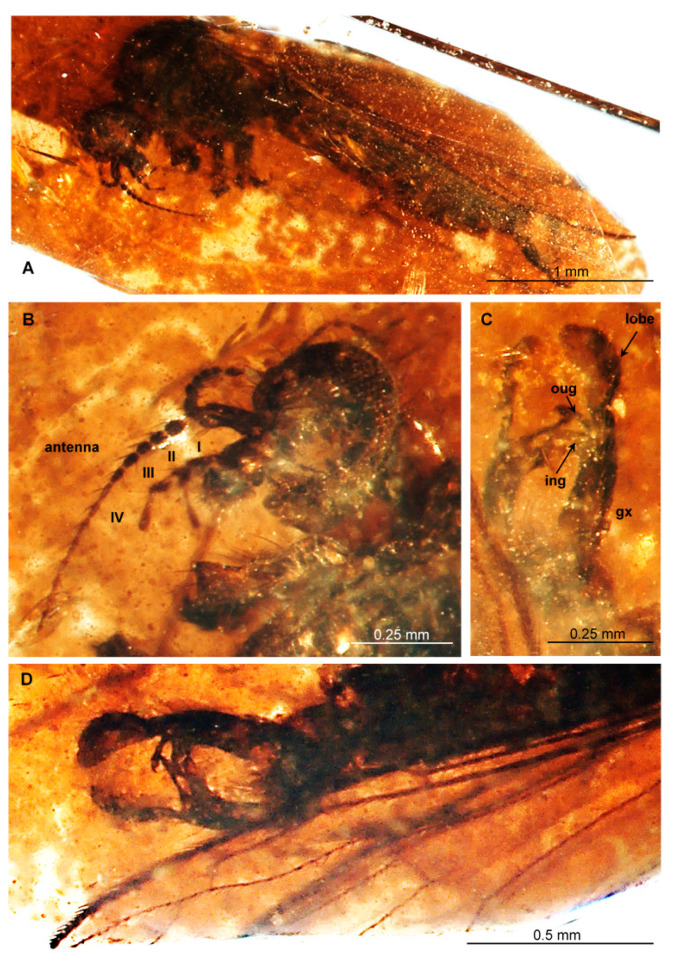
*Trichoneura* (*Cretalinea*) *xavieri* subgen. et sp. nov., holotype No. MCNA 9735 (male). (**A**) body, lateral view; (**B**) head with antenna and palpus visible; (**C**) hypopygium dorsal view; (**D**) hypopygium, ventral view. Abbreviations as in Figure 2.

(Figure 2 and Figure 3)

**Type species**. *Trichoneura* (*Cretalinea*) *xavieri* subgen. et sp. nov., Spanish amber, Lower Cretaceous, upper Albian.

**Etymology.** The subgenus name is derived from “creta” (Latin) = Cretaceous and “linea” (Latin) = line. Gender feminine.

**Diagnosis.** Vein R_1_ terminate in C, opposite approximately 0.8× length of R_2+3+4_; R_3+4_ present, slightly shorter than R_2_ (r-r); bifurcation of Mb before level of the tip of Sc; sc-r shorter than length of section of Sc between sc-r and tip of Sc; gonocoxite with huge, spoon-shaped lobe, which measures almost 0.5× the length of gonocoxite, lobe with relatively short, strong, sparsely distributed setae; outer gonostylus sclerotized, undivided, narrow, only slightly widened at apex, inner and outer gonostylus of comparable length, constitutes less than 0.5× the length of gonocoxite; pronotal appendages absent.

**Comparison.** In contrast to representatives of all other subgenera *Trichoneura*, the subgenus *Cretalinea* subgen. nov. differs by the morphology of the hypopygium, i.e., the presence of a spoon-shaped lobe at the apex of the gonocoxite, with relatively short, strong, sparsely distributed setae. The lobe measuring almost 0.5× the length of the gonocoxite, approximately four times longer than wide, and the gonostylus measuring less than 0.5× the length of the gonocoxite. While the subgenera *Ceratolimnobia* and *Trichoneura* is characterized by a rather short gonocoxite, at most 2.5× as long as wide, and with at least one gonostylus longer than 0.5× the length of the gonocoxite. Moreover, in *Ceratolimnobia* a characteristic process occurs on the outer gonostylus and pronotal appendages are present on antepronotum [20], the pronotal appendages do not occur in *Cretalinea* subgen. nov. Whitish longitudinal lines on the thorax, which occur in *Ceratolimnobia* and are well visible in lateral view, do not occur in the *Cretalinea* subgen. nov. In *Xipholimobia*, the outer gonostylus is deeply bifid, the tip of vein R_1_ is reduced or appears as a short spur [3], whereas in *Cretalinea* subgen. nov. the outer gonostylus is undivided and the tip of R_1_ is well developed, and terminating in C.

**Description.** Body brown, ca. 4 mm long. Head with eyes widely separated. Antenna longer than head, 16-segmented, scape cylindrical, elongate, longer than wide, massive, pedicel small, flagellomeres 3-5 wide and rather short, almost as long as wide; flagellomeres 6–16 elongate, cylindrical, becoming slender to the apex of the antenna; last flagellomere shortest and tiny; flagellomeres 1-15 each with two elongate setae. Maxillary palp four segmented, last palpomere elongate and narrow but widening towards apex.

Wing hyaline, vein Sc not very elongate, terminates before the Rs bifurcation level, beyond the fork of Mb level.

Hypopygium elongate and relatively narrow; gonapophyses small.

**Key to subgenera of *Trichoneura* and fossil species of the genus. Compiled partly after key to fossil species of *Trichoneura* by [9].**

1. Gonocoxite moderate in length, at most 2.5× as long as wide, without lobe at apex; at least one of gonostylus more than 0.5× the length of gonocoxite; outer gonostylus deeply bifid……………………….……………………………………………………………...………. 2.

- Gonocoxite elongate, over 3× as long as wide, with huge lobe at apex, measuring approximately 0.5× the length of gonocoxite; gonostyli measuring less than 0.5× the length of gonocoxite; outer gonostylus undivided ……...………………….…………………………...………………………………………………………. ***T.* (*Cretalinea*) *xavieri*** subgen. et sp. nov.

2. Outer gonostylus deeply bifid …………………………………………………………... 3.

- Outer gonostylus undivided ………………………. ***T.* (*Trichoneura*)** Loew, 1850 [1] 4.

3. Vertex smooth, without corniculus or swelling near anterior end [3]; valves of the ovipositor unequal in length, exceedingly long, slender, a little shorter than the entire remainder of the abdomen [19]; pronotal appendages absent [20] ……………………………...

…………………………………………………………. ***T.* (*Xipholimnobia*)** Alexander, 1921 [5]

Vertex with corniculus or swelling near anterior end [4]; valves moderate length, measuring more than 0.75× the length of entire reminder of the abdomen; pronotal appendages present [20] ………………… ***T.* (*Ceratolimnobia*) *munroi*** Alexander, 1920 [4]

4. Tip of vein R_1_ reduced; vein R_4_ separating from R_2+3+4_ far beyond separation of vein R_2_, and with vein R_3_ forming sector R_3+4_, which measures approximately 0.5× the length of R_3_; cross vein m-cu positioned beyond 0.5× the length of d-cell ………..... ***T.* (*T.*) *canadensis***

- Tip of vein R_1_ well developed, terminating in C; vein R_4_ separate R_2+3+4_ before or at the same point of separation of vein R_2_; cross-vein m-cu positioned before or at 0.5× the length of d-cell ……………………………………………………………………………….… 5.

5. Vein Sc short, terminate opposite 0.75× of Rs; interbase of male genitalia curved at a 90° angle ……………………………………………………………………...… ***T.*** **(*T.*) *wegiereki***

- Vein Sc elongate, terminate opposite approximately 0.85× of Rs or at fork of Rs; interbase of male genitalia straight or slightly arched ………………………………………... 6.

6. Vein R_3_ longer than vein R_2+3+4_; outer gonostylus straight with tip not much wider than the base and provided with straight spine; inner gonostylus bent in the middle at an angle of almost 45°; aedeagus large and thick, nearly as long as the gonocoxite …………………………………………………………………………….…. ***T.* (*T.*) *gracilistylus***

- Vein R_3_ at most as long as R_2+3+4_; outer gonostylus with tip conspicuously widened or narrowed; inner gonostylus only slightly bent; aedeagus small …………………….…. 6.

7. Vein Sc terminating before fork of Rs; R_3_ distinctly shorter than R_2+3+4_; outer gonostylus with strongly widened tip and spine curved inwardly, inner gonostylus with basal 0.3 of its length conspicuously widened and in distal 0.6 of its length strongly narrowed; aedeagus small, about half as long as gonocoxite; ninth tegite broadly excised with a depression in the middle ……………………………………………………………… ***T.*** **(*T.*) *vulgaris***

- Vein Sc elongate, terminating level with fork of Rs; R_3_ subequal or only slightly shorter than R_2+3+4_; outer gonostylus short with very broad base, its distal half strongly narrowed, tip blunt, spineless; inner gonostylus narrowed st 0.3 of its length; aedeagus somewheat longer than the half of gonocoxite …………………………... ***T.*** **(*T.*) *ritzkowskii***

### 3.3. Trichoneura (Cretalinea) xavieri sp. nov.

(Figure 2 and Figure 3)

**Diagnosis.** As for subgenus.

**Etymology.** The specific epithet is dedicated to eminent geologist and paleontologist – Xavier Delclòs from the Universitat de Barcelona, Spain.

***Material examined.** Holotype No. MCNA 9735 (male), Peñacerrada, Álava, Spain*, housed at the Museo de Ciencias Naturales de Álava, Vitoria, Spain.

**Horizon and locality.** The type specimen was found in amber from coal levels with abundant plant remains deposited in delta plain areas that correspond to the top of filling sequences of interdistributary bays. It is also found in filling deposits of abandoned fluvial channels or crevasse splay in the Utrillas Group [15], Lower Cretaceous, upper Albian. The outcrop of Peñacerrada I [21] is located in the Basque-Cantabrian Basin, municipality of Moraza (Province of Burgos, Castilla y León Autonomous Community, northern Spain).

**Description.** Body 3.96 mm long (Figure 2A), brown.

*Head* (Figure 2A,B): antenna (Figure 2A,B and Figure 3A) about 0.97 mm long (1/0.18 mm; 2/0.05 mm; 3/0.06 mm; 4/0.04 mm; 5/0.04 mm; 6/0.05 mm; 7/0.05 mm; 8/0.05 mm; 9/0.04 mm; 10/0.06 mm; 11/0.06 mm; 12/0.06 mm; 13/0.07 mm; 14/0.06 mm; 15/0.04 mm; 16/0.06 mm), scape approximately 3× longer than wide, with a few long and relatively strong elongate setae, longer than width of segments bearing them, 3× longer than pedicel, pedicel only 1.5× longer than first flagellomere, last flagellomere shortest and tiny, 3× longer than wide; flagellomeres 1-15 with two elongate setae, setae on last flagellomere rather short, last flagellomere with two rather short setae arranged at the tip of this segment of antennae. Maxillary palp (Figure 2A,B and Figure 3B) elongate, first and third palpomeres equal in length, 2× longer than wide, widened in distal part; second palpomere shortest, 1.5× longer than wide; last palpomere elongated and narrow, approximately 7× longer than wide, widened in apical part, length of palpomeres 0.47 mm: 1/0.12; 2/0.09 mm; 3/0.14 mm; 4/0.12 mm.

Wing (Figure 2A,D and Figure 3C): Rs as long as R_2+3+4_ and R_3+4_ combined; fork of R_3+4_ distal to R_1_ insertion into C.

Hypopygium (Figure 2C,D and Figure 3D): elongate and relatively narrow, 0.63 mm long, gonocoxite 0.40 mm long, inner gonostylus 0.14 mm, outer gonostylus 0.12 mm, lobe on hypopygium 0.23 mm long with relatively short, strong, sparsely distributed setae; inner gonostylus approximately twice width of outer gonostylus, process structure with two short setae at apex 0.5x the length of inner gonostylus, measuring 0.6x width of inner gonostylus.

Remarks. The specimen is not well preserved. Wings of the specimen are not clearly visible, and are only partially preserved. The legs are almost completely destroyed. The hypopygium is well preserved and the most important taxonomic features of the species are visible.

## 4. Discussion

**Figure 4:**
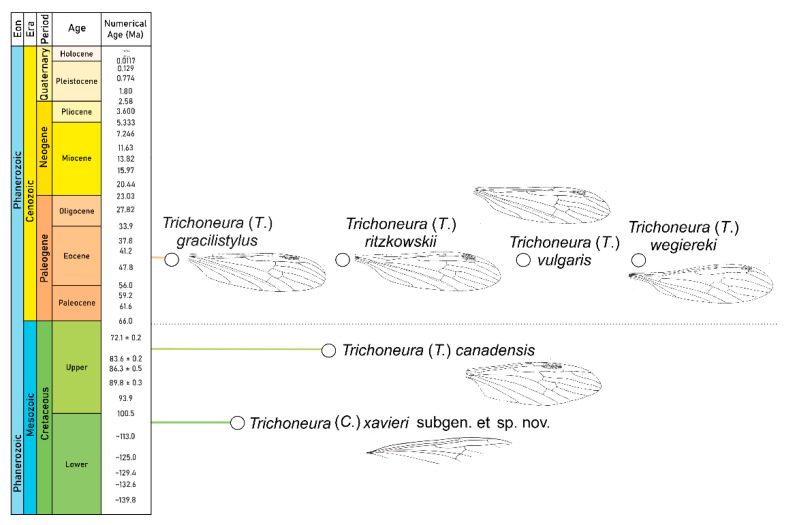
Chronostratigraphic distribution of *Trichoneura*, fossil species*,* redrawn after [1,6,7,8,9]. Chronostratigraphic chart and the colors of the lines that indicate age of extinct species of *Trichoneura* are used according to International Chronostratigraphic Chart v.2020.03 (https://stratigraphy.org/chart) (accessed on 29 April 2021).

**Figure 5:**
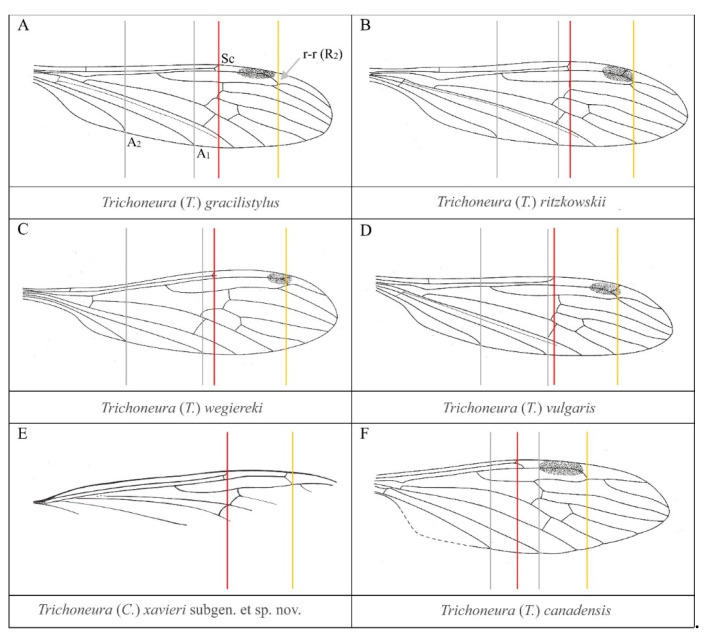
Wing venation of extinct species of the genus *Trichoneura.* Abbreviation: vertical lines indicate the line ends – grey lines tips of A_1_ and A_2_; red line, tip of Sc; yellow tip of R_1_. The drawings are redrawn after [1,6,7,8,9].

Deposits of Spanish amber were formed in the Lower Cretaceous period in upper Albian later than the deposits of Lower Cretaceous Lebanese amber (lower Barremian [22,23]) and earlier than the Burmese amber deposits (Upper Cretaceous, Cenomanian [23]. Among the inclusions originating from this period there are many significant transformations of fauna and flora. Some of the oldest members of the subfamily Limoniinae e.g., the oldest representatives of the genus *Helius* Lepeletiere et Serville, 1928 [24] have been found in Lebanese amber – such as *Helius ewa* Krzemiński, Kania et Azar, 2014 [25] and *Helius lebanensis* Kania, Krzemiński et Azar, 2013 [26]. From Spanish amber, only three species of Limoniidae have been recorded until now: *Alavia neli* Krzemiński et Arillo, 2007 [27], *Helius alavensis* Kania, Krzemiński et Arillo, 2016 [28], and *Helius spiralensis* Kania, Krzemiński et Arillo, 2017 [29]. Added to these the newly described species—*T.* (*Cretalinea*) *xavieri* subgen. et sp. nov.—the oldest representative of the genus and new subgenus (Figure 4). The genus *Trichoneura* has so far been represented in the fossil record only by one subgenus, *Trichoneura*. In recent fauna, the subgenus *Trichoneura* is represented by only by one species, while the subgenus *Xipholimnobia* is the richest in species but is not present in the fossil record. There are more extant than fossil species, although the subgenus *Ceratolimnobia* is represented by only one species – *Trichoneura* (*Ceratolimnobia*) *munroi* (Alexander, 1920) [4]. Subgenera differ mainly in the morphology of the hypopygium and wing venation. The diversity of wings of fossil species belonging to the family and representatives of each subgenus are shown in Figure 5. There are clearly visible differences in the presence or reduction of the terminal sector of vein R_1_, and development of R_3+4_ as in *T.* (*T.*) *canadensis* (Figure 5). Five species from the fossil record, including a new species, *T.* (*Cretalinea*) *xavieri* subgen. et sp. nov., have obvious R_1_. Due to the fact that there is a tendency to reduce the end of R_1_, e.g. in Tipulidae Latreille, 1802 [30], it can be supposed that the well-developed R_1_ end is a plesiomorphic feature, and is more often characteristic of fossil species than modern ones. However, in *Trichoneura* this section of venation is variable, R_1_ is sometimes obsolete, appearing as short spur. 

**Figure 6:**
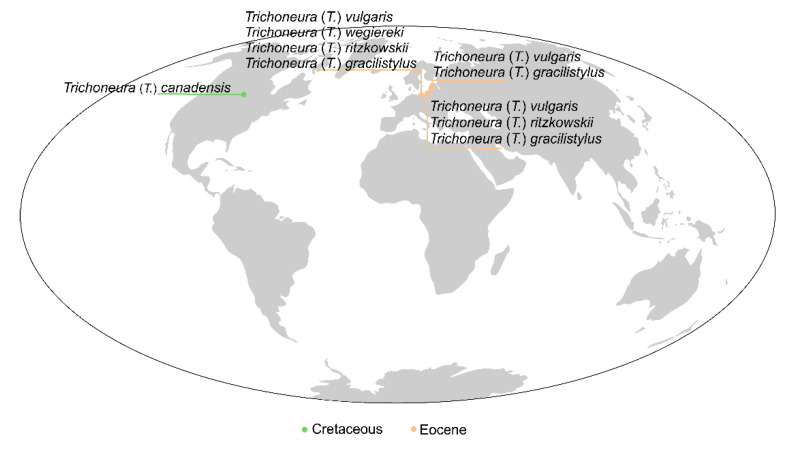

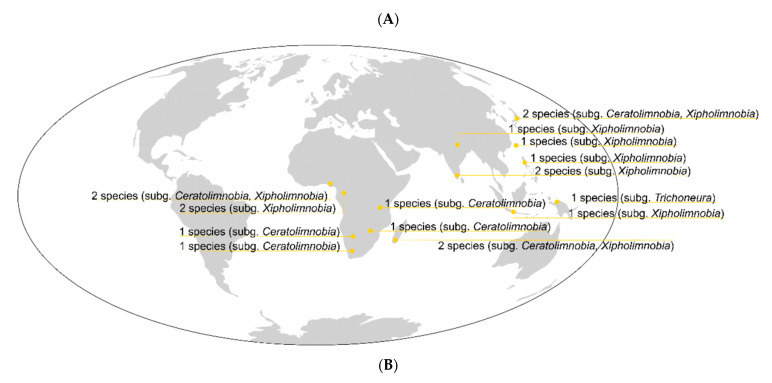
(**A**) Map of the distribution of fossil localities of the representatives of the genus *Trichoneura.* (**B**) Map of the distribution of the representatives of the genus *Trichoneura* in recent fauna (the data provided according to [2]). A. the colors of the lines that indicate fossil localities are used according to International Chronostratigraphic Chart v.2020.03 (https://stratigraphy.org/chart) (accessed on 29 April 2021).

It is also worth noting that all fossil species of the genus *Trichoneura*, both those known from the Cretaceous and the Eocene, have been found in the northern hemisphere, while most of the modern species classified to the genus occur in the southern hemisphere. This may be related to the climate, because deposits of resins such as Lebanese were formed in tropical or subtropical, moderate to hot and very wet, dense forests in the north-east of Gondwana [31], while Baltic amber deposits were formed during warming episode, in the Middle Eocene Climatic Optimum (MECO), the wormest period in Earth’s history [32]. Today, representatives of the genus *Trichoneura* are species associated with warmer areas on Earth including parts of Africa and Southeast Asia [2] (Figure 6).

## 5. Conclusions

*Cretalinea* subgen. nov. shows a unique morphology of hypopygium in which a relatively large, spoon-shaped lobe occurs measuring almost half the length of the gonocoxite. This particular structure of hypopygium is currently known only from one place and time, the Cretaceous Spanish amber period, and probably did not survive to recent times. This Cretaceous evolutionary lineage of *Trichoneura* provides the possibility to change the chronostratigraphic distribution of the genus from the late Cretaceous to the early Cretaceous. The newly discovered species is the oldest representative of *Trichoneura* and sheds new light on the evolution of this genus.

## Figures and Tables

**Table 1:** List of species of *Trichoneura* previously known from fossil record.

Species	Time Scale	Type of Material	Locality
*Trichoneura* (*Trichoneura*) *gracilistylus* Alexander, 1931 [7]	Eocene	Baltic amber	Baltic area
*Trichoneura* (*Trichoneura*) *ritzkowskii* Krzemiński, 1990 [8]	Eocene	Baltic amber	Baltic area
*Trichoneura* (*Trichoneura*) *wegiereki* Kania, 2015 [9]	Eocene	Baltic amber	Baltic area
*Trichoneura* (*Trichoneura*) *vulgaris* Loew, 1850 [1]	Eocene	Baltic amber	Baltic area
*Trichoneura* (*Trichoneura*) *canadensis* Krzemiński et Teskey, 1987 [6]	Upper Cretaceous	Canadian amber	Canada

## Data Availability

Data is contained within the article.
